# Combined x-ray crystallography and computational modeling approach to investigate the Hsp90 C-terminal peptide binding to FKBP51

**DOI:** 10.1038/s41598-017-14731-z

**Published:** 2017-10-27

**Authors:** Rajnish Kumar, Martin Moche, Bengt Winblad, Pavel F. Pavlov

**Affiliations:** 1 Department of Neurobiology, Care Sciences and Society, Center for Alzheimer Research, Division of Neurogeriatrics, Karolinska Institutet, Huddinge, Sweden; 2 Department of Medical Biochemistry and Biophysics, Protein Science Facility, Karolinska Institutet, 171 77 Stockholm, Sweden; 3 Department of Geriatric Medicine, Karolinska University Hospital, Huddinge, Sweden

## Abstract

FK506 binding protein of 51 kDa (FKBP51) is a heat shock protein 90 (Hsp90) co-chaperone involved in the regulation of steroid hormone receptors activity. It is known for its role in various regulatory pathways implicated in mood and stress-related disorders, cancer, obesity, Alzheimer’s disease and corticosteroid resistant asthma. It consists of two FKBP12 like active peptidyl prolyl isomerase (PPIase) domains (an active FK1 and inactive FK2 domain) and one tetratricopeptide repeat (TPR) domain that mediates interaction with Hsp90 via its C-terminal MEEVD peptide. Here, we report a combined x-ray crystallography and molecular dynamics study to reveal the binding mechanism of Hsp90 MEEVD peptide to the TPR domain of FKBP51. The results demonstrated that the Hsp90 C-terminal peptide binds to the TPR domain of FKBP51 with the help of di-carboxylate clamp involving Lys272, Glu273, Lys352, Asn322, and Lys329 which are conserved throughout several di-carboxylate clamp TPR proteins. Interestingly, the results from molecular dynamics study are also in agreement to the complex structure where all the contacts between these two partners were consistent throughout the simulation period. In a nutshell, our findings provide new opportunity to engage this important protein-protein interaction target by small molecules designed by structure based drug design strategy.

## Introduction

Molecular chaperones are primarily responsible not only for the maintenance of intracellular protein homeostasis including protein folding, transport and degradation but also are involved in variety of specific functions including stress response, intracellular signaling, and transcription^1^. The action of molecular chaperones heat shock protein 70 (Hsp70) and heat shock protein 90 (Hsp90) is mediated by the co-chaperones: proteins interacting with molecular chaperones and providing specificity to their reactions in the cell. Together they form a highly organized network responsible for the intracellular homeostasis also enabling quick cell adaptation to various stimuli. The mechanism of interaction of one particular group of co-chaperones containing tetratricopeptide repeat (TPR) motif with Hsp70 and Hsp90 is well understood^2^. TPR domain represents repeats of 34 amino acid TPR-motif and in FK506 binding proteins (FKBPs) there are three TPR-motifs and a single additional helix making up 7 anti-parallel alpha helices as a TPR domain^3^. Out of 736 TPR motif-containing proteins annotated in the human UniProt database, approximately 20 different proteins interact with Hsp70 and Hsp90 via “di-carboxylate clamp” mechanism^2,4^. Hsp70 and Hsp90 localized in cytosol contain C-terminal amino acid sequences -Glu-Glu-Val-Asp (EEVD) that are necessary and sufficient for their interaction with the TPR domain of co-chaperones. Often TPR proteins possess modular structure where various functional domains such as protein phosphatase^5^, E3-ubiquitin ligase^6^, or immunophilin^7^ are connected to TPR domain that mediates their interaction with their partner proteins. The immunophilins, which include both the FKBPs and the cyclophilins (Cyp), are also targets of immunosuppressive drugs FK506 (tacrolimus), rapamycin (sirolimus) and cyclosporine A (CsA). Immunophilins exhibit peptidyl prolyl cis/trans isomerase (PPIase) activity in the cell facilitating protein folding^7^. Several immunophilins including Cyp40, FKBP52, FKBP51, FKBP38, FKBP37, also known as aryl hydrocarbon receptor interacting protein (AIP), and FKBP36 interact with Hsp70/Hsp90 molecular chaperones via their TPR domains suggesting evolutionary importance of functional co-operation in protein folding. Several studies have suggested that modulation of Hsp90 or Hsp70 may be clinically relevant for protein mis-folding disorders^8–11^. Identification of co-chaperones that specifically affect protein subclasses (i.e. transmembrane receptors or unstructured proteins) could provide more specific drug targets with fewer adverse consequences.

FKBP51, originally discovered as a component of steroid receptor complexes, is now known to regulate a diverse set of transcription factors, enzymes and structural proteins. FKBP51 is the preferred TPR immunophilin for mature glucocorticoid receptor (GR)-Hsp90 complexes and represses GR function, with FKBP51 over-expression resulting in a receptor with decreased corticosteroid sensitivity^12–14^. Cellular properties of FKBP51 suggest its numerous possible physiological functions related to steroid hormone signaling. For example, PPIase activity of FKBP51 towards proline-rich tau protein promotes tau stability in cytosol^15^. Down-regulation of FKBP51 expression in cell lines or deletion of *FKBP5* gene in mice resulted in reduction of tau intracellular levels^15,16^. Strategies aimed at attenuating FKBP51 levels or its interaction with Hsp90 have the potential to be therapeutically relevant for Alzheimer’s disease (AD) and other tauopathies^16^. In line with the role of FKBP51 in stress hormone response, studies in FKBP51 knockout mice revealed an increased resistance to stress stimuli, significantly lower levels of stress hormones in blood after the stress resulting in mice anti-depressive behavior^17^. Studies in mice provided evidence for the role of FKBP51 in stress-related disorders^18–21^, chronic pain control^22^ as well as in regulation of metabolism^23^. Numerous human genetic association studies also implicated FKBP51 into mood and stress related mental disorders^24^. The association of FKBP51 with stress-related disorders and neurodegenerative diseases, together with the protective effects seen with FKBP51 depletion in animal models of depression and anxiety make this co-chaperone a promising drug target.

Recently, selective inhibitors of PPIase activity of FKBP51 have been developed. They exhibit greater than 10000 folds selectivity towards FKBP51 over its highly similar homolog FKBP52 and have shown anti-depressive and anti-anxiety effect in treated mice^25^. Inside the cell, the TPR domain of FKBP51 mediates interaction with EEVD C-terminal sequence of Hsp90/Hsp70 molecular chaperones. Considering the critical role of EEVD motif in the interaction as well as relatively small interaction area between the TPR domain and the EEVD peptide, it should be possible to disrupt the chaperone-co-chaperone interactions. Indeed, Yi and Regan *et al*.^26^, have identified small organic compounds inhibiting Hsp90 interactions with TPR co-chaperone HOP. However, the issue of selectivity of these molecules towards other TPR co/chaperones of Hsp90/Hsp70 has not been investigated.

Use of computational drug design tools in early phase drug discovery has grown rapidly owing to their high speed and low cost^27,28^. Structure based drug design (SBDD) can be employed as a tool to identify small molecule inhibitors of this interaction taking the advantage of the availability of the 3D crystal structures of FKBP52^29^, FKBP38^30^, FKBP37/AIP^31^ and Cyp40^32^ in complex with the EEVD peptides. These crystal structures reveal that the TPR domain binds to the Hsp70/Hsp90 C-terminal peptide in more or less similar fashion which makes the task of identifying selective inhibitors of a specific co-chaperone very challenging. The crystal structure of FKBP51 has been solved without the interacting MEEVD peptide^33^ therefore, as a first step towards SBDD to identify novel specific inhibitors of FKBP51/Hsp90 interaction, it was important to solve the structure of FKBP51 in complex with the MEEVD peptide. In the present study, we have performed x-ray crystallography study to solve the structure of the FKBP51-Hsp90 C-terminal peptide complex and get insight into their interactions. Molecular dynamics simulations have also been successfully employed to analyze and visualize the time evolution of the complex. Additionally, in order to understand the binding in detail, we performed biophysical characterizations as well. We believe that the FKBP51-Hsp90 C-terminal peptide complex structure reported in this study can be instrumental in design and development of novel specific and potent small molecule inhibitors of this important drug target. However, it worth to mention that some of the functions of FKBP51 might not be dependent on its interaction with Hsp90 and will not be affected by such inhibitors.

## Results

### Biophysical characterization and determination of dissociation constant under thermal denaturation using differential scanning fluorimetry

**Figure 1:**
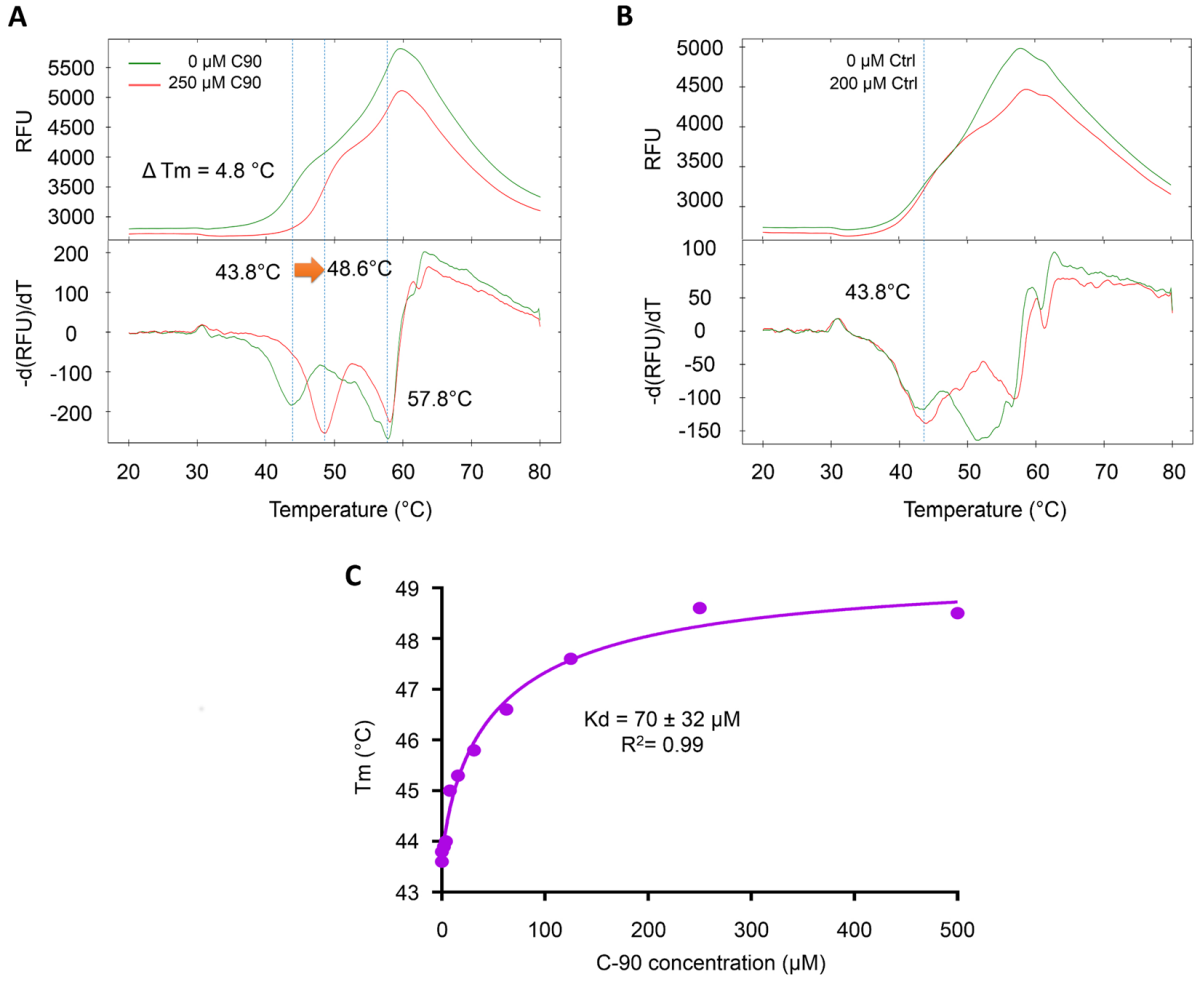
Biophysical characterization of C90 peptide binding to the FKBP51 TPR domain by DSF analysis. (**A**) DSF melting curve and first derivative curve of the FKBP51 with 0 µM (green line) and 250 µM C90 peptide (red line). The upper half of the figure shows the melting curve and the lower half shows the first derivative curve to determine the melting point. Major multiple transitions occurred at 43.8 and 57.8 °C for FKBP51 in the absence of C90 peptide. In the presence of 250 µM of C90 peptide the first melting point shifted to 48.6 °C without much change in the second melting point. A ΔTm of 4.8 °C indicates specific biding of C90 peptide to the TPR domain of FKBP51. (**B**) DSF melting curve and first derivative curve of the FKBP51 with 0 µM (green line) and 200 µM control peptide (DDDDDDDDDD) (red line). The upper half of the figure shows the melting curve and the lower half shows the first derivative curve to determine the melting point. The presence of peptide did not change the melting point indicating that it is not interacting with FKBP51. (**C**) Binding affinity of C90 peptide to TPR domain is determined by incubation with increasing (0–250 µM) concentrations of C90 peptide.

The differential scanning fluorimetry (DSF) is a widely used, quick and inexpensive method to determine and evaluate the thermal stability of protein and can also be used as a method to detect the interaction between two partners of the interaction^34^. It is also possible to use this method for rapid and inexpensive determination of affinity of the protein to its interacting partners^35^. The DSF spectrum of the FKBP51 possesses multiple meting transition which occurred between 44 and 58.6 °C in the absence of Hsp90 C-terminal peptide containing sequence as “HHHHHHDTSRMEEVD” (C90) peptide (Fig. 1A). The presence of multiple melting transitions may be ascribed to the distinct unfolding events of different domains of FKBP51. It is evident from its structure that the FK1, FK2 and TPR domains are independent of each other and may unfold independently. In the presence of C90 peptide the first melting point shifted to 48.6 °C without affecting the second melting point. A ΔTm of 4.8 °C is observed which suggests that this peak is arising from unfolding of the TPR domain. It also indicates specific binding of C90 peptide to the TPR domain of FKBP51. Additionally, we have used control peptide NH2-DDDDDDDDDD-COOH to ensure that the binding to the TPR domain of FKBP51 is sequence-specific and not a simple electrostatic attraction between peptide and its target. We observed that the incubation of FKBP51 with 200 µM control peptide did not lead to any change in the melting point of FKBP51 (Fig. 1B). Further, a full screen at different concentrations of C90 was performed and the Tm values were recorded using the first derivative method. The obtained data were analyzed using model for a single binding event and resulted in kd value of 70 ± 32 µM with an R^2^ value of 0.99 (Fig. 1C).

### Crystal structure of FKBP51 in complex with C90 peptide

**Figure 2:**
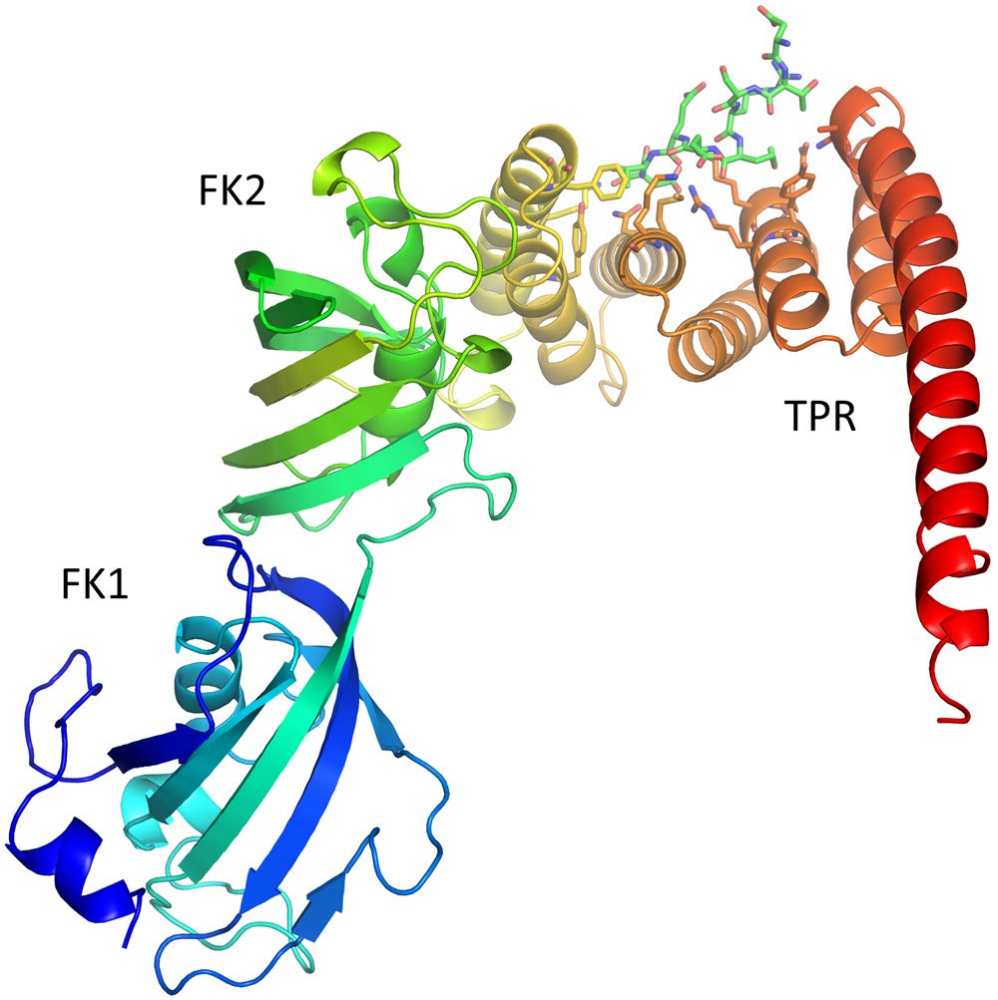
Crystal structure of FKBP51-Hsp90 C-terminal peptide complex. The overall structure of the complex contains FK1, FK2 and TPR domains. The C90 peptide is shown as sticks with carbons in green color.

Crystal structures of native FKBP51 (PDB ID: 5OMP) and FKBP51 in complex with the C90 peptide (PDB ID: 5NJX) were determined to understand their interaction (Fig. 2). Going from the N-terminus to C-terminus, the overall FKBP51 structure contains two mixed beta sheet wrapped around a central helix for FK1 and FK2 domains followed by the TPR domain consisting of three TPR repeats and one additional long helix in the C-terminus. FK1 domain is active as PPIase and known to bind to the FK506 and rapamycin inhibitors or modulators^36^. The TPR domain is protein-protein interaction site with a concave surface which hosts the C-terminal peptide from Hsp90.

The x-ray dataset were anisotropic and therefore, scaled using diffraction anisotropy server and the Staraniso software respectively, for the complex structure and the native structure. The elongated shape of FKBP51 leads to non-uniform crystal packing and therefore anisotropic order and diffraction from our crystals. For the native, we used reflections up to 1.88 Å in elliptical shape with 93% completeness (52% completeness with spherical truncation) while standard spherical truncation would have resulted in a 2.45 Å resolution being 100% complete. Similarly, for the complex structure we obtained a resolution of 2.49 Å with 62% spherical completeness. The native structure has R and R_free_ values of 0.198 and 0.247, respectively at the resolution of 1.88 Å (52% spherical completeness) while the complex structure has the R and R_free_ values of 0.192 and 0.262, respectively at 2.49 Å (62% spherical completeness).

The data and refinement statistics for both of the structures is given in Table 1. The final resolution for native FKBP51 and its complex with Hsp90 peptide was 2.55 and 2.15 Å, respectively.

**Table 1:** Data collection and refinement statistics.

	FKBP51- peptide complex	FKBP51
**Data collection**		
Space group	P 32 2 1	P 32 2 1
Cell constants		
a, b, c (Å)	92.16, 92.16, 132.01,	90.73, 90.73, 133.59,
α, β, γ (°)	90, 90, 120	90, 90, 120
Ellipsoidal truncation software	UCLA anisotropy server	Staraniso
Resolution (Å)	44.0–2.49 (2.55–2.49)	133.6–1.88 (2.15–1.88)
% Spherical data completeness	62.3 (2.5)	52.1 (8.0)
% Ellipsoidal data completeness^1^	not calculated by UCLA	93.2 (74.8)
Rmeas	0.128 (0.756)	0.096 (1.60)
I/σ(I)	19.3 (3.8)	20.3 (1.9)
Multiplicity	19.3 (17.0)	19.4 (17.0)
Beamline	BESSY BL14–1	DIAMOND i04–1
**Refinement**		
No. reflections all/free (%)	14 504/729 (5.03%)	27 108/1314 (4.85%)
R, Rfree	0.192, 0.262	0.198/0.247
Fo, Fc correlation	0.908/0.838	0.942/0.910
Refinement software	BUSTER 2.10.3	BUSTER 2.10.3
Wilson B-factor (Å^2^)	57.4	42.7
Average B, all atoms (Å^2^)	61.3	57.7
No. atoms/Average B (Å^2^)		
Protein	3352/62	3291/58
Sulfate	15/104	15/73
Water	175/35	348/53
R.m.s. deviations		
Bond lengths (Å)	0.008	0.01
Bond angles (°)	1.08	1.10
Ramachandran Plot		
Favoured (%)	93.9	97.8
Allowed (%)	5.2	2
Disallowed (%)	0.9	0.2
PDB id	5NJX	5OMP

^1^Both datasets were elliptically truncated by either the UCLA diffraction anisotropy server (https://services.mbi.ucla.edu/anisoscale/) for 5NJX or Staraniso software for 5OMP.

Our model is more complete and contains less gaps as compared to the previous reported structure^33^ of native FKBP51 that contained 357 amino acid residues as compared to our model which contains 413 amino acid residues out of total 457. Additionally, our model is very similar to the reported structure and the root mean square deviation (RMSD) is 0.62 Å for 356 aligned residues using COOT^37^. Using secondary-structure matching (SSM) server^38^, we compared the RMSD between our two structures and previously reported structure and found that the RMSD between native and the complex structure was 0.48 Å and the RMSD between native and previously reported structure was 0.49 Å while the RMSD between the complex and reported structure was found to be 0.61 Å for 349 aligned residues. The two native structures are more similar as compared to the complex structure. We also compared our structure with the squirrel monkey FKBP51 (1KT0) being 94% sequence identity with the human FKBP51. The average RMSD between squirrel monkey FKBP51 as compared to human FKBP51 is 0.90 and 0.84 Å, respectively for complex and the native structures.

FKBP51 has been seen in a complex with FRB domain of human mTOR and in our native structure we noticed that the Arg73 is not in the same position as in the FKBP51-FRB complex (PDB ID:4DRI)^36^. This particular arginine is missing in the previously reported structure of FKBP51 and the corresponding arginine residue in FKBP12 is Arg42 Mutation studies of this Arg42 changed to lysine and isoleucine resulted in conformational changes leading to disorientation of FKBP12 and FK506 which interacts with calcineurin. These conformational changes lead to reduced calcineurin inhibition by FKBP12 yet retaining full PPIase activity^39^.

**Figure 3:**
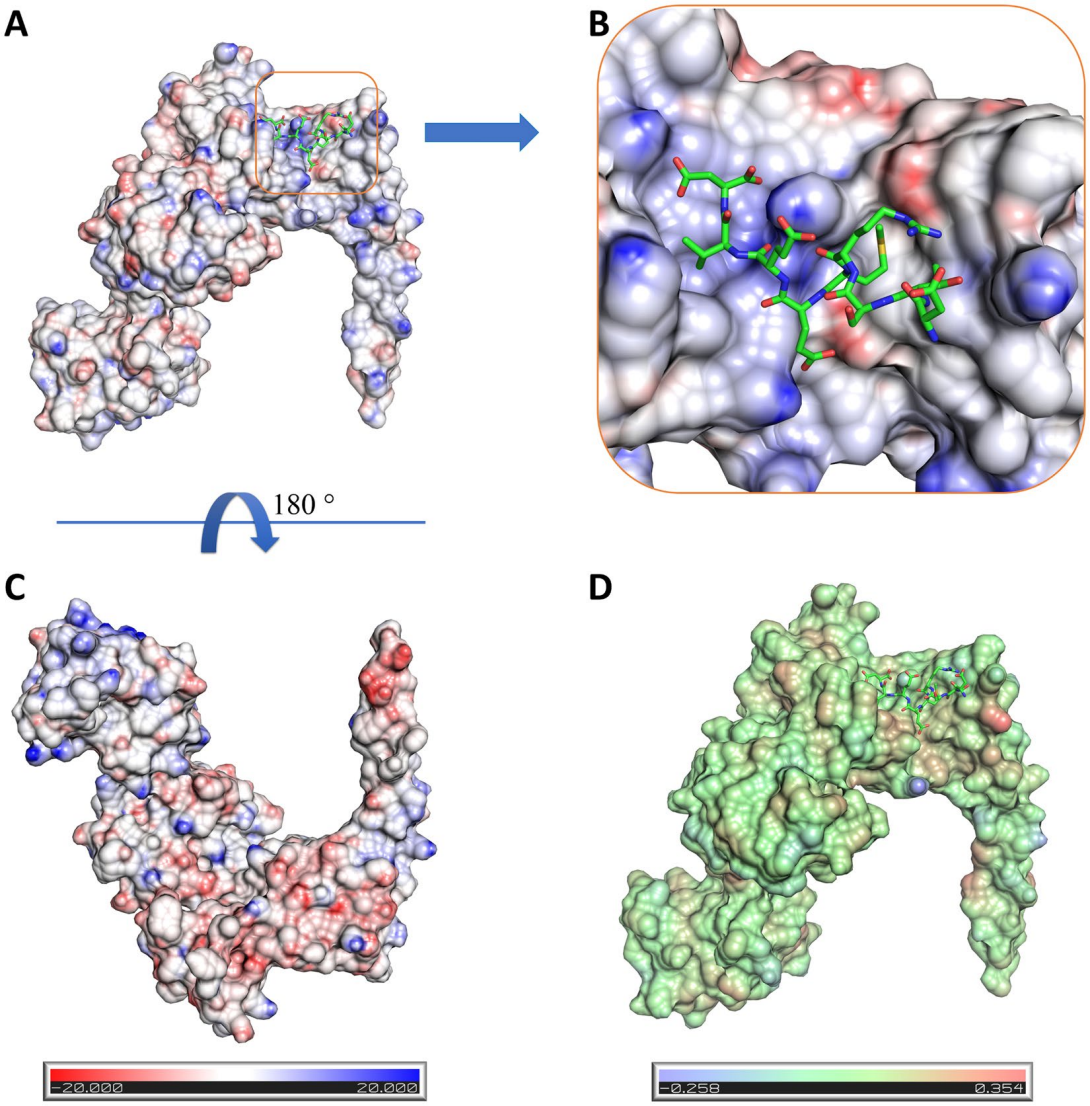
Surface characteristics of the FKBP51 structure. (**A**) Electrostatic surface potential of FKBP51. (**B**) Zoomed-in view of the peptide binding pocket. The binding pocket is electropositive in nature and thus facilitates the binding of electronegative peptide. (**C**) +180° rotated view around x-axis. (**D**) Surface hydrophobicity of the FKBP51.

We run the Vasco software that enables to display the electrostatic and hydrophobic surface and observed that in the complex surface the concave groove in the TPR domain is positively charged which makes long range attractions for the negatively charged C90 peptide (Fig. 3A–C). The hydrophobic surface was also drawn using Vasco and is shown in Fig. 3D.

### Validation of the crystal structure

**Figure 4:**
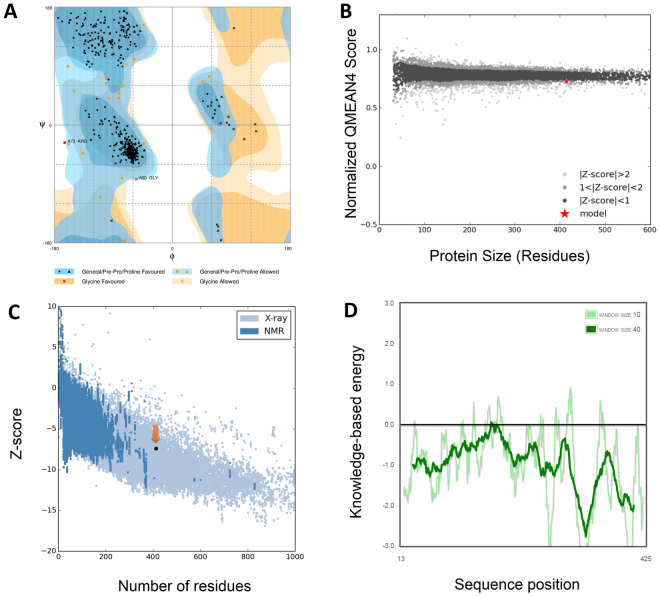
Validation of the FKBP51 model by multiple methods. (**A**) Ramachandran plot. (**B**) Qmean based structure validation which compares the structure to non-redundant set of PBDs of similar size. The FKBP51 structure indicated as a red star lies within the range of scores of similar size structures, indicating its good quality. (**C**) ProSa Z-score plot of the FKBP51 indicated by red arrow. (**D**) The local quality of the model indicated as plot of energy as a function of amino acid sequence position.

The quality of crystal structure coordinates was validated using several methods. The Ramachandran analysis^40^ illustrated that 395 (94.5%) residues are in favored region and 21 (5%) are in allowed region. Only 2 (0.5%) residues are in outlier region (Fig. 4A). Using the Qualitative Model Energy Analysis (QMEAN) server^41^, the model quality was determined. The overall quality of model was good as indicated by its QMEAN Z-score and QMEAN4 global score (Fig. 4B). Low quality models are expected to have a negative QMEAN Z-score. The QMEAN4 ranges from 0 to 1 and a higher value indicates good quality model^41^. Additionally, the overall quality of the model was evaluated using Protein Structure Analysis (ProSA) tool (https://prosa.services.came.sbg.ac.at/prosa.php) which provides a quality score, Z-score as compared to all known protein structure from x-ray crystallography as well structural NMR^42^. The obtained Z-score value was −7.38 which indicates the high quality of the model compared to known protein structures (Fig. 4C). The local quality of the model was also calculated and is presented in Fig. 4D.

### FKBP51-Hsp90 C-terminal peptide interaction

**Figure 5:**
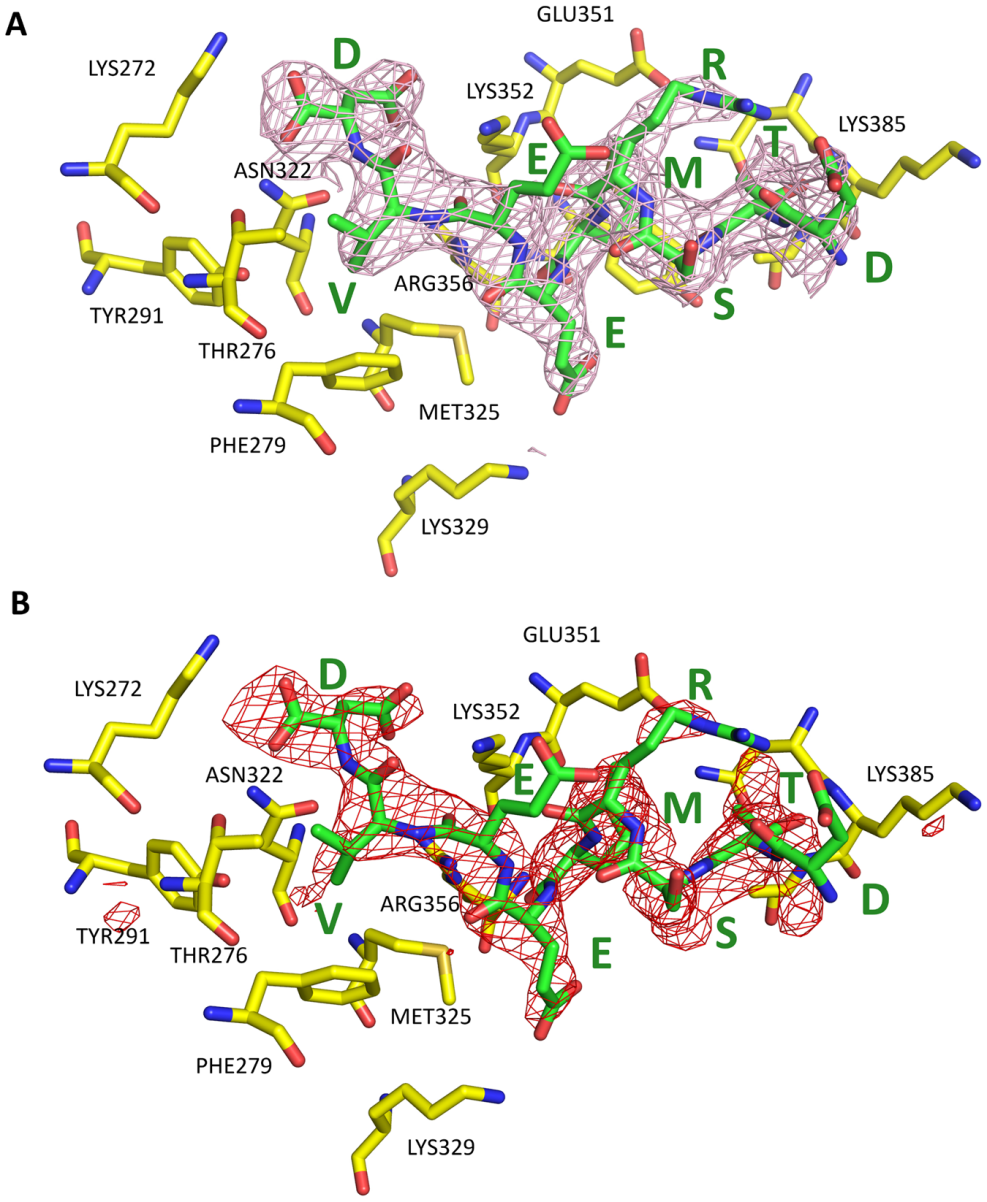
Electron density maps of the Hsp90 C-terminal peptide binding to the TPR domain of FKBP51. (**A**) The sigma weighted 2fo-fc electron density map calculated by Buster (contoured at 1.2 σ) of Hsp90 C-terminal peptide bound to the TPR domain of FKBP51. (**B**) The sigma weighted simulated annealing omit map (contoured at 3 σ) of Hsp90 C-terminal peptide bound to the TPR domain of FKBP51.

**Figure 6:**
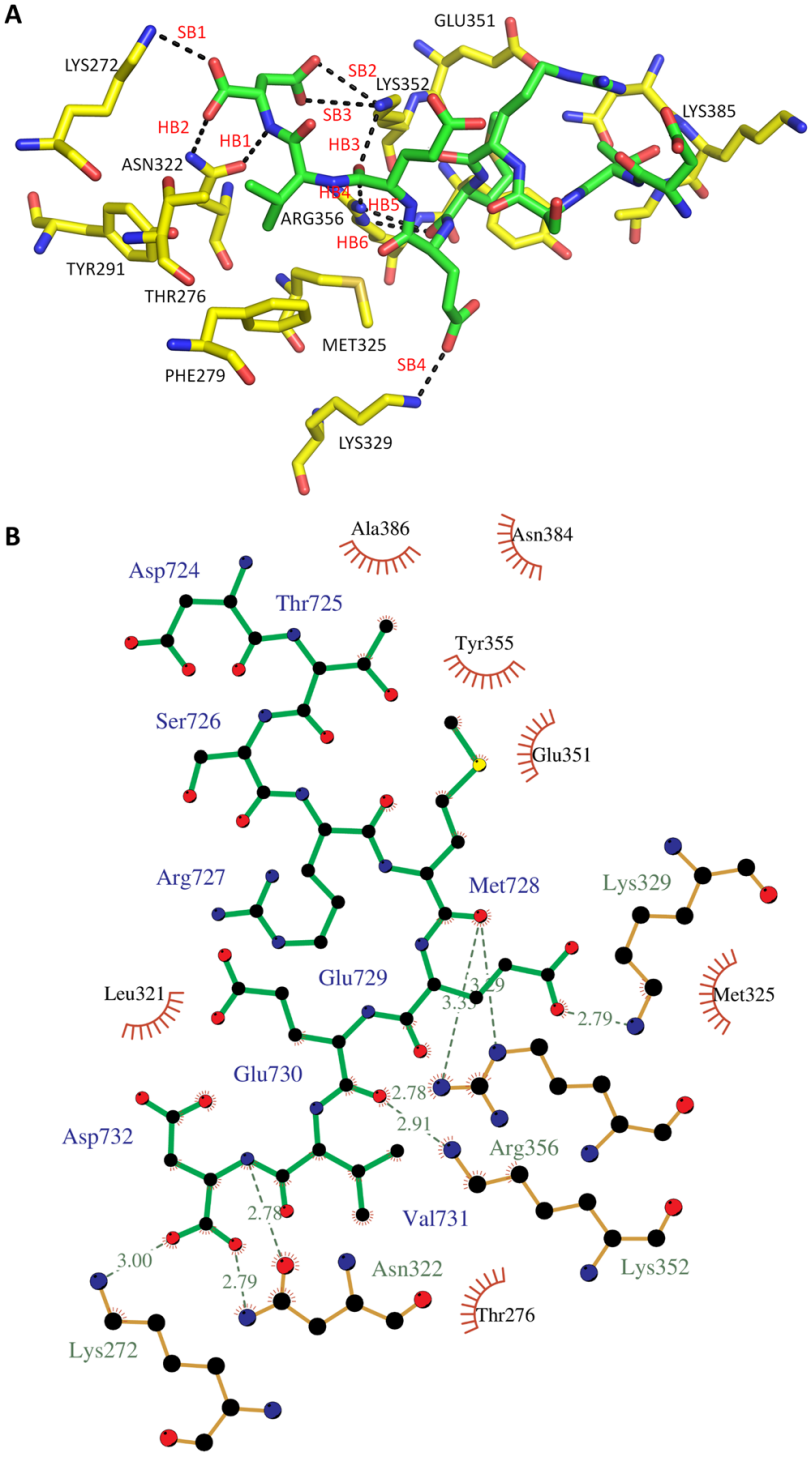
Interaction of Hsp90 C-terminal peptide with the TPR domain of FKBP51. (**A**) Hydrogen bond and salt bridge network of C90 peptide bound to the TPR domain of FKBP51. Yellow carbon sticks shows TPR domain and the MEEVD peptide is shown as green carbon sticks. Hydrogen bonds and salt bridges are represented as black dotted lines and are labeled. The figure is generated by PyMOL. (**B**) 2D ligand interaction diagram created by LigPlot+ showing the hydrogen bond network and the hydrophobic interactions of the peptide with the TPR domain of FKBP51.

The overall structure of the FKBP51-Hsp90 DSTRMEEVD peptide complex with the TPR domain of the FKBP51 is shown in Fig. 5A. Interestingly, we observed that out of 9 amino acids, all except one are involved in some kind of interactions with the TPR binding pocket (Tables 2 and 3). The valine and methionine side chains of the C90 peptide are buried into hydrophobic pockets contributing to the complex formation (Table 3). It is also observed that the C-terminal aspartate (Asp732) is involved in formation of the di-carboxylate clamp with the help of hydrogen bonding and salt bridges. Hydrogen bonds and salt bridge output from PISA server (http://www.ccp4.ac.uk/pisa/) is given in Table 2. PISA uses a cut-off distance of 3.89 Å between the donor and acceptor for hydrogen bonding and 4 Å between heavy atoms for salt bridge^43^. However, for our analysis we used a hydrogen bond cut-off of 3.6 Å as suggested by reported literature^44^. The results indicated that the Asp732 nitrogen formed a hydrogen bond with the OD1 atom of carboxylate group of Asn322 with a distance of 2.78 Å (Fig. 6A). Another hydrogen bond was formed at a distance of 2.79 Å involving OXT atom of Asp732 and ND2 atom of Asn322. Asp732 is also interacting to Lys272 and Lys352 with the help of three salt bridges SB1, SB2 and SB3 with a length of 3, 3.39 and 3.70 Å, respectively. The fourth salt bridge SB4, is formed between Glu729 and Lys329 at a distance of 2.79 Å. Glu730 was involved in formation of hydrogen bonds, HB3, HB4, HB5, and HB6 with Lys352 and Arg356 with a length of 2.91, 2.78, 3.33 and 3.29 Å, respectively. Additionally, the 2D interaction diagram showing hydrogen bonds network and the hydrophobic interactions created by LigPlot+^45^ is shown in Fig. 6B. According to UniProt database the TPR domain can be divided into three repeats, TPR1 comprising of residues from Ala268 – Glu301, TPR2 comprises of Leu317-Asn350 and TPR3 contains Glu351-Asn384 amino acid residues. All these three subdomains of TPR are involved in binding of the peptide.

**Table 2:** Hydrogen bonds and salt bridges in the FKBP51-C90 peptide complex.

Hydrogen bonds	C90 peptide	TPR	Length (Å)	Average length during MD simulation (Å)
HB1	B:ASP_732_[N]	A:ASN_322_[OD1]	2.78	3.3 ± 0.6
HB2	B:ASP_732_[OXT]	A:ASN_322_[ND2]	2.79	3.8 ± 1.0
HB3	B:GLU_730_[O]	A:LYS_352_[NZ]	2.91	3.7 ± 1.1
HB4	B:GLU_730_[O]	A:ARG_356_[NH2]	2.78	3.3 ± 0.6
HB5	B:MET_728_[O]	A:ARG_356_[NH2]	3.33	3.4 ± 0.5
HB6	B:MET_728_[O]	A:ARG_356_[NE]	3.29	4.1 ± 0.7
Salt Bridges				
SB1	B:ASP_732_[O]	A:LYS_272_[NZ]	3.00	3.6 ± 0.8
SB2	B:ASP_732_[OD1]	A:LYS_352_[NZ]	3.39	3.6 ± 0.9
SB3	B:ASP_732_[OD2]	A:LYS_352_[NZ]	3.70	3.5 ± 0.9
SB4	B:GLU_730_[OE1]	A:LYS_329_[NZ]	2.79	8.9 ± 2.6

**Table 3:** Interface residues information for C90 peptide.

Residue	IS	HS	ASA	BSA	Delta G
B:ASP_724_	I		167.45	5.34	0.02
B:THR_725_	I	H*	90.62	72.82	0.39
B:SER_726_	s		71.45	0.00	0.00
B:ARG_727_	I		123.80	31.56	−0.24
B:MET_728_	I	H	152.27	145.75	2.61
B:GLU_729_	I	S	153.26	75.80	−0.53
B:GLU_730_	I	H	116.31	23.47	−0.12
B:VAL_731_	I		143.06	110.03	1.74
B:ASP_732_	I	HS	198.83	133.32	−0.68

I = interface residue, s = surface residues, H = hydrogen bond, S = Salt bridge, ASA = Accessible surface area (Å^2^), BSA = Buried surface area (Å^2^), Delta G = hydrophobic effect in Kcal/mol, *Hydrogen bonds longer than 3.6 Å are not included in our analysis according to reported literature^44^.

### Comparison to other structures binding to Hsp90 C-terminal peptides

**Figure 7:**
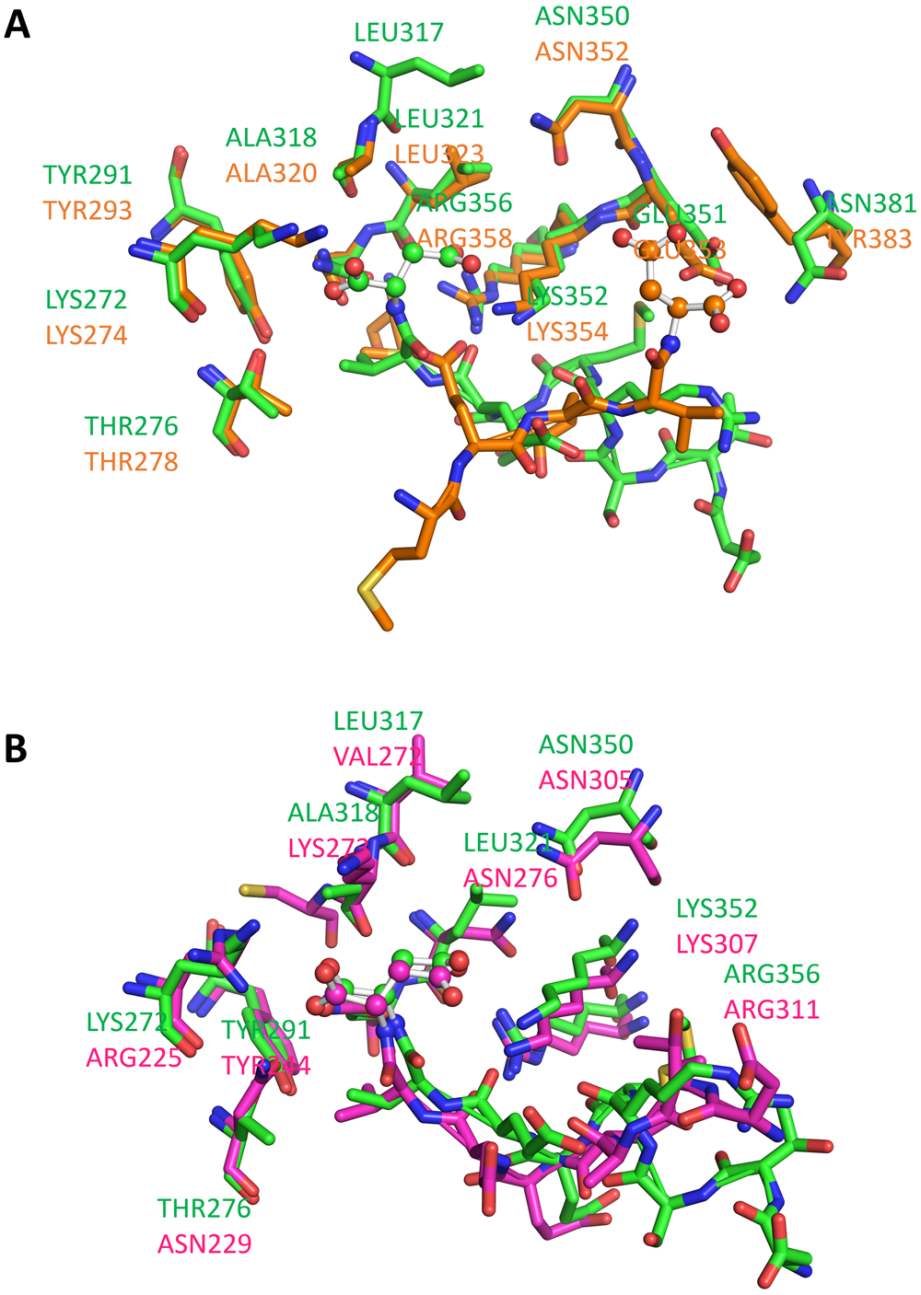
Comparison of binding of MEEVD peptide to the FKBP51 vs FKBP52 (**A**) and FKBP51 vs FKBP38 (**B**). The C-terminal Asp amino acid is rendered as ball and stick.

Many members of the FKBP family such as FKBP51, FKBP52, FKBP37 and FKBP38 have the TPR domain which is important for interaction with the Hsp90 and Hsp70 peptide. The TPR domain of FKBP51 has a sequence identity of 59% to FKBP52 while only 30% and 22% to FKBP37 and FKBP38, respectively. Therefore, the MEEVD peptide is expected to bind in a similar fashion to FKBP51 and FKBP52. However, when comparing our structure to the FKBP52-MEEVD peptide complex (1QZ2) it does not match despite very high conservation of the binding pocket amino acid side chains (Fig. 7A). In the case of FKBP51 vs FKBP38, we observed a similar binding peptide conformation despite low amino acids conservation between these two proteins (Fig. 7B).

When calculating the electron density using the deposited coordinates (1QZ2) and the structure factors, we could not generate the convincing electron density maps for the peptide in the FKBP52-peptide complex. We believe that the peptide structure in the complex is not modeled correctly as observed earlier^30^. Our own peptide electron density maps are reasonable as presented in Fig. 5A and B. The Glu730 side chain has no electron density (Fig. 5) which might be due to its flexibility in the absence of any hydrogen bonding or salt bridges with the TPR binding pocket residues (Table 3).

### Conservation analysis of TPRs

**Figure 8:**
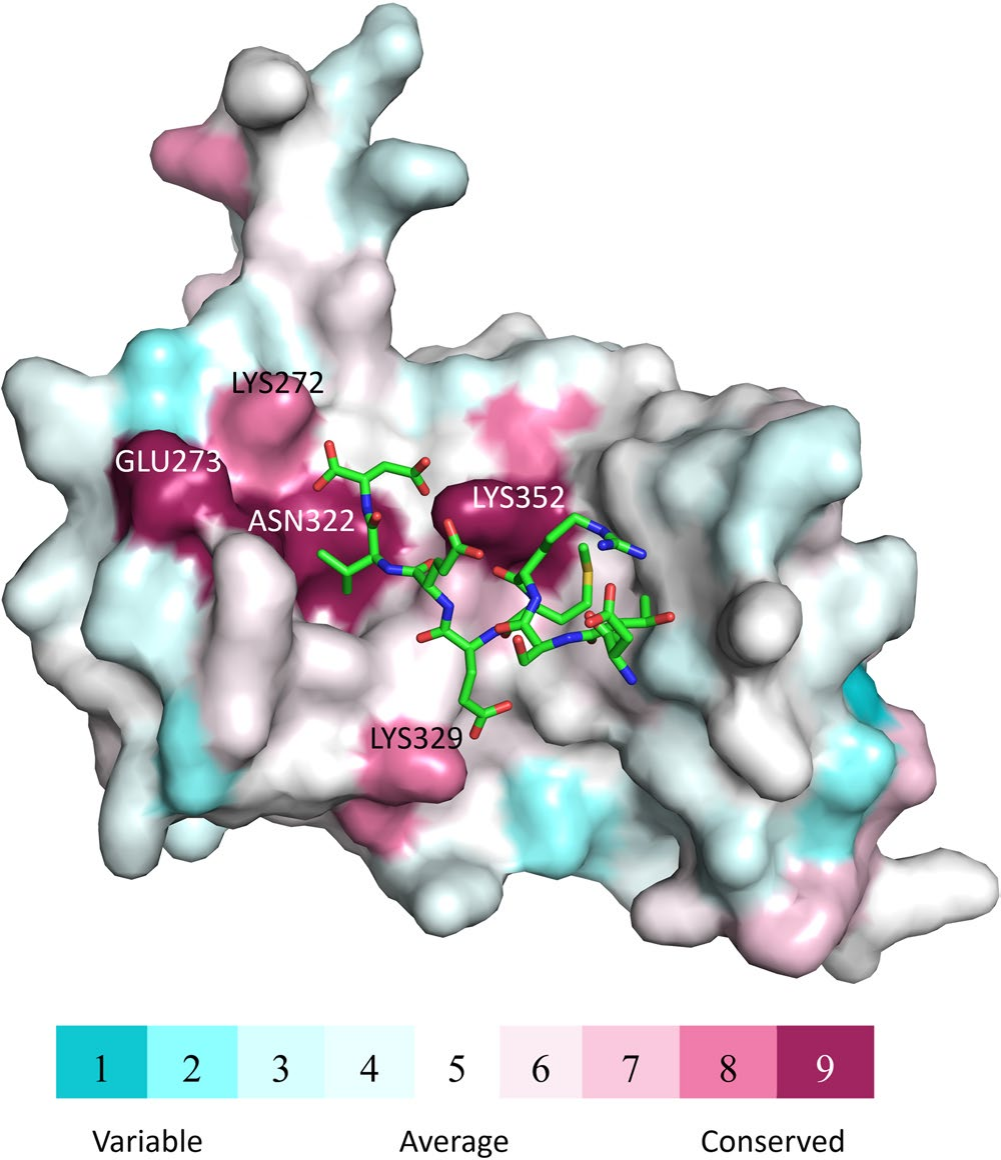
ConSurf analysis for the TPR domains of TPR-containing proteins. The 3D structure of TPR domain of FKBP51 is rendered as surface and the bound C90 peptide is rendered as sticks. The TPR domain’s surface is color coded by its conservation grade using the color-coding bar shown in the figure, with turquoise-through-maroon indicating the variable to conserved residues. The figure indicates that the functionally important amino acid residues, taking part in the binding to the C90 peptide are highly conserved. The analysis was carried out using the solved crystal structure and the figure was generated with the help of PyMOL script output by ConSurf.

ConSurf is a bioinformatics tool which provides evolutionary conservation profile of protein structures. It is well known that the function of a protein depends on its structure and during the evolution functional amino acid residues in the proteins remained highly conserved. The final result from the conservation analysis using ConSurf for the selected TPR domains is shown in Fig. 8. The final scores are color coded and the maroon color indicates high conservation while white and turquoise colors indicate average and very low conservation, respectively. The results indicated, as expected, that the functional part of TPR domain is highly conserved. For example, the Lys352 which is largely involved in formation of H-bonds and salt bridges with the C90 peptide and Lys272 which forms a salt bridge with the terminal Asp732 of the Hsp90 peptide are highly conserved. Lys329 forming a salt bridge with the Hsp90 peptide is also highly conserved. Additionally, Asn322 and Glu273 also showed high conservation score which indicates that they might provide additional environment to the formation of di-carboxylate clamp and helps the C90 peptide to bind strongly to the TPR binding pocket.

### Molecular dynamics simulation

**Figure 9:**
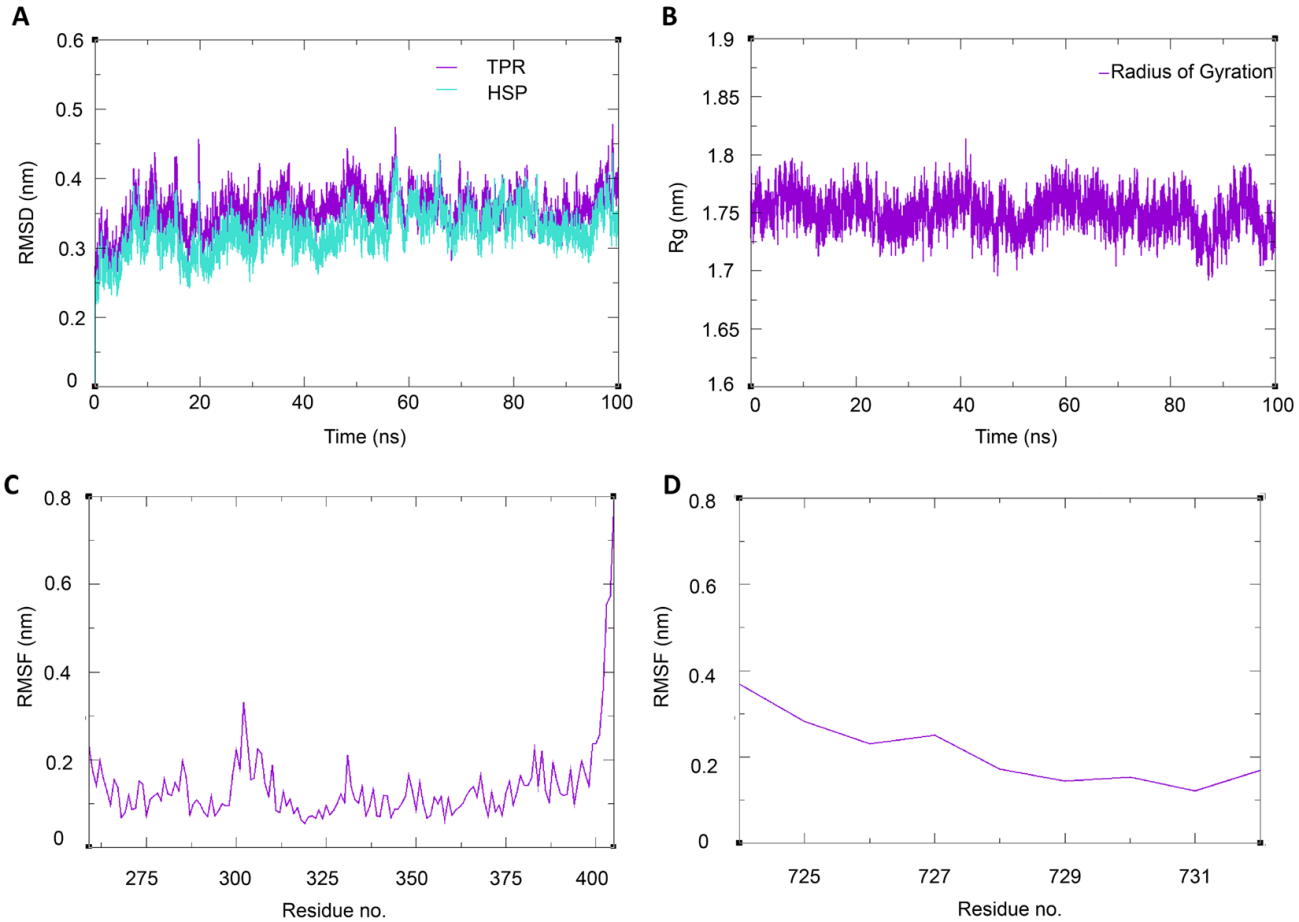
Qualitative analysis of the Molecular dynamics trajectory. RMSD plot of TPR domain of FKBP51 and C90 peptide (**A**). Radius of gyration of the complex (**B**). RMSF of TPR (**C**) and RMSF of C90 peptide (**D**).

We carried out conventional molecular dynamics simulation of the experimentally determined structure of the FKBP51-C90 complex. The fluctuation of the structure of simulated complex from its initial coordinates gives important information about the stability and quality of the system. As given in Fig. 9A, at the starting of the trajectory the backbone RMSD value was around 0.3 nm which indicates that the small fluctuations in the structure occurred during the NVT and NPT equilibration steps but later the RMSD changes were very small indicating the well-stabilized TPR-C90 complex during 100 ns simulation. Further, radius of gyration of the Cα atoms provided a measure of overall compactness of the complex (Fig. 9B). Additionally, in order to evaluate the flexibility of the complex throughout the simulation, we calculated RMSF of individual amino acid residues of the complex. As it can be seen from the Fig. 9C, in the TPR domain residues 302–305, which forms a small loop to connect the helixes showed higher flexibility than the rest of the structure. The terminal residues of the TPR domain also showed very high flexibility. In case of the C90 peptide the Asp732 residue which is involved in formation of the di-carboxylate clamp showed less flexibility indicating that this residue works as an anchor in the complex (Fig. 9D **)**. On the other hand the Asp724 showed higher flexibility as anticipated.

**Figure 10:**
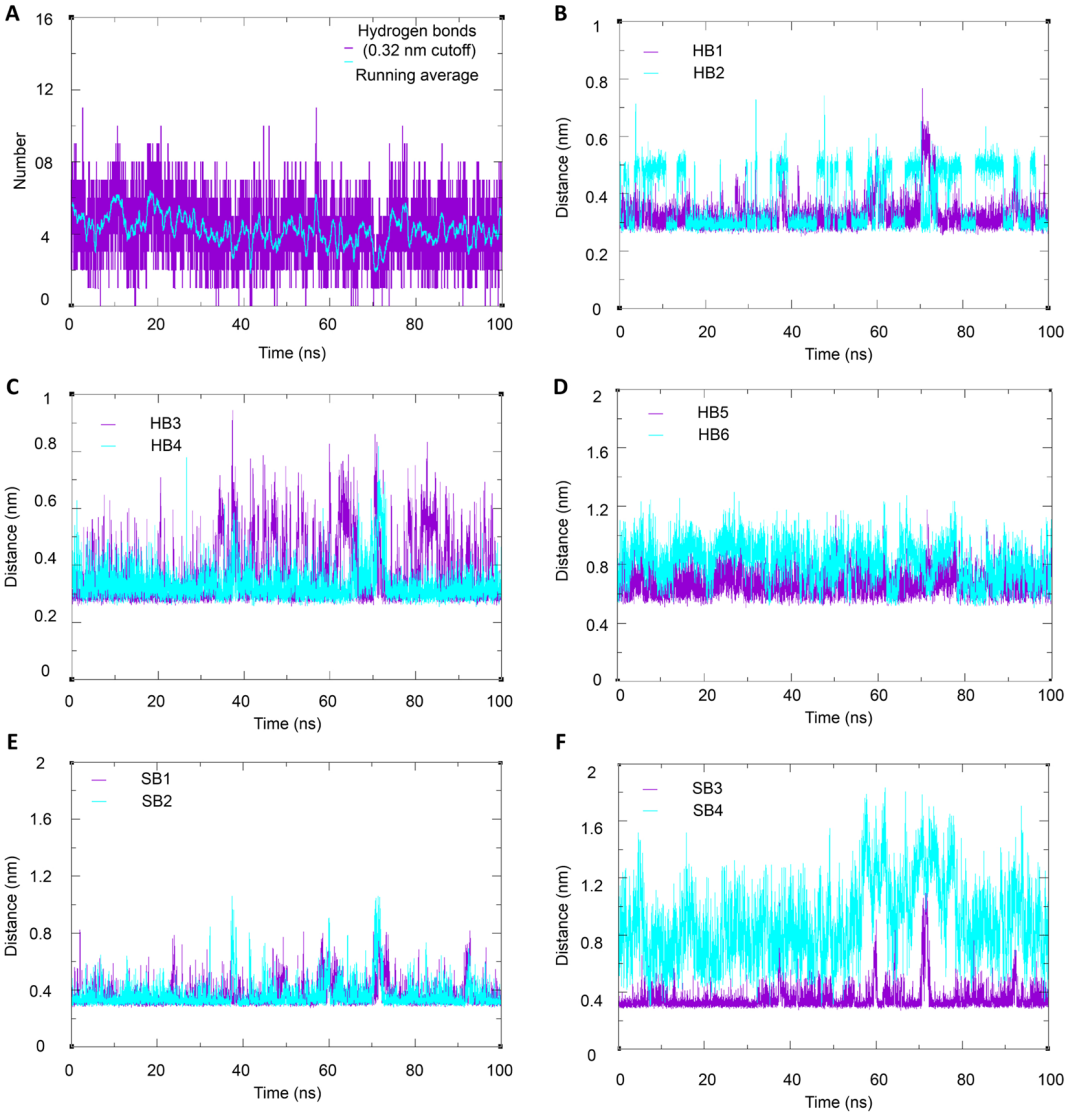
Hydrogen bond analysis of the molecular dynamics trajectory. Total number of hydrogen bonds formed during 100 ns simulation trajectory (**A**). Length of HB1 and HB2 (**B**). Length of HB3 and HB4 (**C**). Length of HB5 and HB6 (**D**) Length of SB1 and SB2 (**E**) and Length of SB3 and SB4 (**F**).

Hydrogen bonding, salt bridges, and hydrophobic interactions play a central role in the formation and stability of protein complexes. During the simulation, all the hydrogen bonds and the salt bridges which were present in the crystal structure (obtained from the jsPISA server) were traced. The average number of hydrogen bonds calculated with a cut-off value of 3.2 Å using the h-bond utility in GROMACS is given in the Fig. 10A. It was observed that most of the hydrogen bonds and salt bridges obtained in the crystal structure were retained throughout the simulation trajectory. The HB1-HB6 has an average length of 3.3 ± 0.6, 3.8 ± 1.0, 3.7 ± 1.1, 3.3 ± 0.6, 3.4 ± 0.5, and 4.1 ± 0.7, respectively and are plotted in Fig. 10B–D. It was observed that all the HBs were in the acceptable range of hydrogen bond distance. Similarly, the average length of salt bridges for SB1, SB2, SB3, and SB4 were 3.6 ± 0.8, 3.6 ± 0.9, 3.5 ± 0.9, and 8.9 ± 2.6 Å, respectively and are plotted in Fig. 10E and F. The length of SB4 increased unexpectedly during the molecular dynamics simulation. Keeping in mind that the current structure contains C-terminal peptide of Hsp90 and not a full length structure the observed instability of SB4 may indicate the complexity of interaction of the Hsp90 with the TPR domain of FKBP51 and thus might need solving the crystal structure of full length Hsp90 and FKBP51 protein complex^33^.

**Figure 11:**
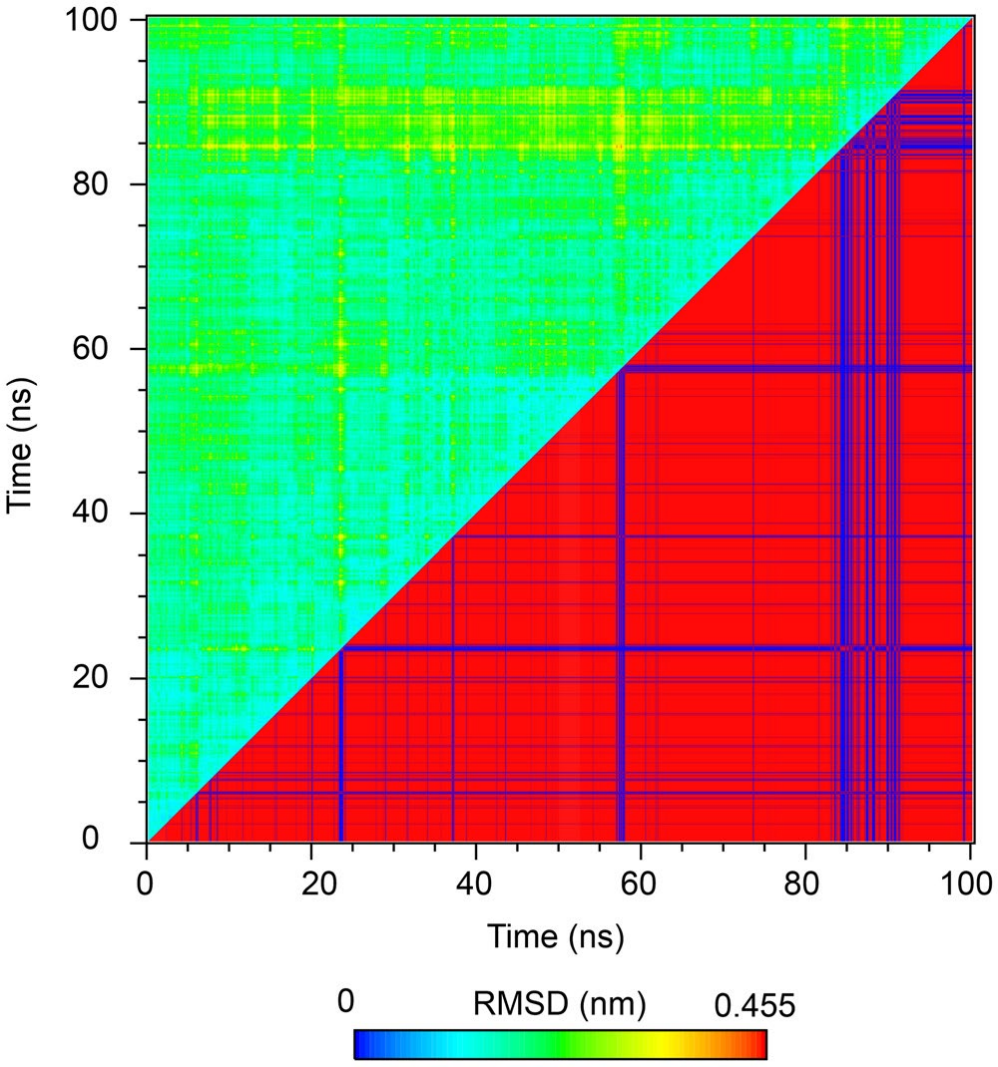
Cross-RMSD matrix of the backbone. In the upper left half the coordinates belonging to same clusters (red) and different cluster (blue) represented in the lower half.

**Figure 12:**
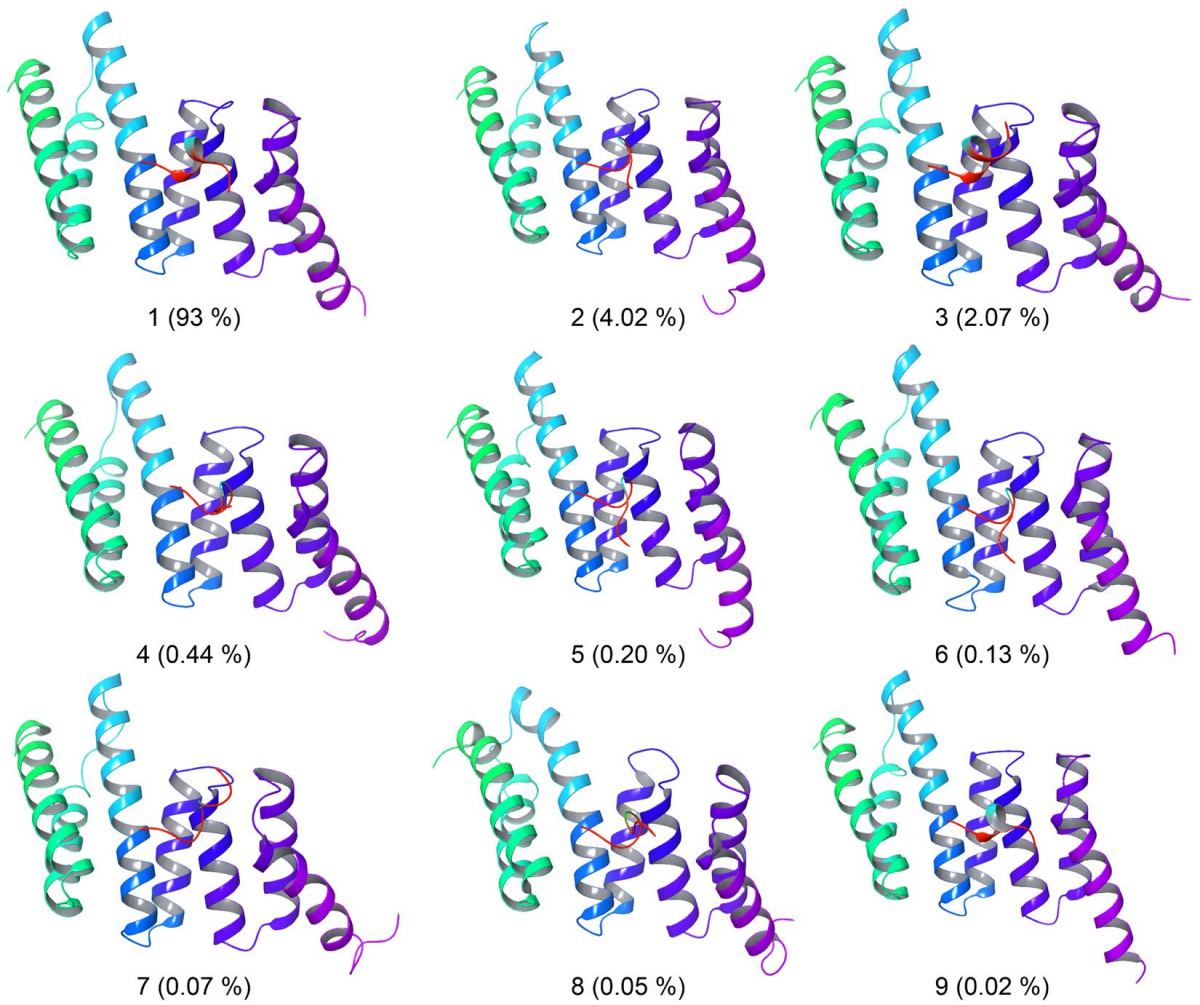
Median structures of the clusters obtained after MD analysis. The size of cluster is given in the parenthesis as percentage population.

Figure 11 show the matrix of pairwise RMSDs. Very few transitions were observed marked by high RMSD value near the diagonal. Overall the RMSD did not change rapidly throughout the simulation representing a stable complex structure. Furthermore, using a cut-off value of 0.2 nm and gromos clustering algorithm, we obtained 9 clusters. The average RMSD values ranged from 0.05 to 0.46 nm and the average RMSD was 0.11 nm. The representative structure from each cluster is presented in Fig. 12 along with their % population. Clusters 1, 2 and 3 were the largest clusters containing 93, 4.02 and 2.07% of overall trajectory structures. The other smaller clusters 4–9 contained only 0.44, 0.20, 0.13, 0.07, 0.05, and 0.02% of all the trajectory structures, which indicated that the cluster 1 dominates throughout the simulation period. It is also interesting to observe that the Asp732 of C90 peptide remained anchored to the TPR domain of FKBP51 (Fig. 12) while the other end is more flexible and contributes to the different population of structures.

## Discussion

FKBP51 is the co-chaperone to Hsp90 and belongs to the immunophilin family initially recognized for its involvement in regulation of steroid hormone receptor activity. Lately it has been reported to be involved in regulation of prostate and glioma cancers^46^, pain^47^, Alzheimer’s disease and taupathies^16^, stress-related disorders^18–21^ and corticosteroid resistant asthma^48^ which makes it an interesting target for development of drugs against these diseases. As an important step toward understanding the mechanism of binding of Hsp90 C-terminal peptide to the TPR domain of FKBP51, we, in this study characterized the molecular level details underlying the complex formation between FKBP51 and C90 peptide using a combination of differential scanning fluorimetry, x-ray crystallography and molecular dynamics simulation.

Differential scanning fluorimetry is a well-established biophysical method to characterize proteins stability and can also be used as a rapid and cost-effective method to determine binding affinity of ligand to the proteins. We used this versatile method to determine the dissociation constant of FKBP51-C90 complex under thermal denaturation. Most commonly the value of dissociation constant calculated using this method is slightly higher than the one observed using isothermal titration calorimetry or surface plasmon resonance because of the fact that the dissociation constant is positively correlated to the temperature of the system^35^. However, the dissociation constant value for the complex is very similar to the reported dissociation constant of FKBP38-MEEVD peptide^30^.

We solved the crystal structure of both the native form of FKBP51 and in complexation with the C90 peptide. During x-ray data collection we noticed that the diffraction pattern was quite anisotropic and therefore, decided to truncate the data in an ellipsoidal fashion using diffraction anisotropy server for the complex and the Staraniso software for the native structure. While doing ellipsoidal truncation to keep the good data, the consequence is that the completeness in the higher resolution shells is getting very low as compared to standard spherical truncation. The ellipsoidal truncation had positive impact on the electron density maps and on the refinement software used for making the best possible models. The structure of native FKBP51 is already reported at a resolution of 2.7 Å^33^. However, our main interest was to solve the structure of the complex for further use in the structure based design of FKBP51-C90 interaction inhibitors.

Intriguingly, the structure of the complex was similar to other complexes of MEEVD peptides with TPR domains such as FKBP38-MEEVD peptide complex^30^ indicating the importance and conservation of di-carboxylate clamp as an essential part of the complex formation. Unfortunately, we could not see any differential binding pattern between Hsp90 and Hsp70 C-terminal peptide binding to the TPR domains. Since the structure of FKBP52-Hsp90 MEEVD peptide complex is not correct as discussed in a latest publication^30^, we were not able to conclude the differences between binding of Hsp90 C-terminal peptide to FKBP51 and FKBP52. The conservation profile of TPR domains of several di-carboxylate clamp TPR containing proteins indicated that the important amino acid residues which take part in hydrogen bonding and salt bridge formation such as Lys329, Glu273, Asn322, Lys272 and Lys352 are highly conserved as evident from the current results and from the reported literature as well.

Since crystal structures represent a still picture of the proteins complexes at action, we, therefore, performed molecular dynamics analysis of the structure obtained from x-ray crystallography to obtain time evolution of the complex. Notably, a 100 ns simulation trajectory revealed that the complex structure remains essentially stable while preserving the hydrogen bonds and salt bridges observed in the crystal structure. In particular, the final clustering of molecular dynamics trajectory coordinates gave one large cluster containing 93% of overall structures which indicated that the FKBP51-C90 peptide complex remained stabilized throughout the molecular dynamics simulation.

In summary, the combination of x-ray crystallography and molecular dynamics approaches has allowed us to investigate the binding of C90 peptide to the TPR domain of the FKBP51. Our results suggested that the peptide binds in a similar fashion through the di-carboxylate clamp as in the case of Hsp70/Hsp90 peptide binding to the other TPR domain- containing proteins. We believe that the results from this study could open a new avenue for the structure based design of novel small molecule inhibitors of FKBP51 for engaging this highly attractive drug target.

## Materials and Methods

### Peptide synthesis

The NH_2_-HHHHHHDTSRMEEVD-COOH peptide consisting of N-terminal 6xHis-tag attached to sequence corresponding to the last nine amino acids of human Hsp90 alpha has been synthesized at GL Biochem. Shanghai, China.

### Protein expression and purification

FKBP51 full length DNA (IMAGE cDNA clone: 5753397 5′) was PCR amplified with forward and reverse primers containing BamHI and XhoI overhangs and cloned into pGEX6–1 vector (GE Healthcare, Uppsala, Sweden) at BamHI and XhoI restriction sites. Insert was sequence verified and the plasmid was transformed into BL21 *E*.*coli* strain. Gst-FKBP51 was purified from 1 L overnight culture after 2 h induction with 1 mM IPTG. Cells were pelleted, re-suspended in 1x PBS and sonicated 3 × 20 sec on ice. PMSF, EDTA and protease inhibitor cocktail was added to prevent proteolysis. The suspension was centrifuged for 30 min 50.000 × g to remove cell debris and supernatant was loaded onto 1 ml Gst-trap 4B column (GE Healthcare Uppsala, Sweden). After column washing with 30 ml PBS Gst-FKBP51 was eluted with 2.5 ml 10 mM Gluthatione in PBS. Eluate was passed through PD-10 column (GE Healthcare, Uppsala, Sweden) to remove free gluthatione. Gst-FKBP51 was cleaved at 4 °C overnight with PreScission protease (GE Healthcare, Uppsala, Sweden) and passed through Gst-trap column to remove free Gst protein. Average yield from 1 L starting culture was 5 mg of FKBP51. Protein containing fractions were collected, checked with SDS-PAGE and kept at −20 °C.

### Determination of dissociation constant using differential scanning fluorimetry

Differential scanning fluorimetry was performed using Bio-Rad CFX96 qPCR following a well-established protocol^34^. Briefly, 25 µl of solution containing purified FKBP51 at a concentration of 0.1 mg/ml, 5X SYPRO orange (Life Technologies), and Hsp90 C-terminal peptide ranging from 0–500 µM in 0.5X PBS was transferred to a 96-well PCR plate in triplicate. The plate was centrifuged, sealed and the fluorescence was measured using the FRET channel between 20 and 80 °C with an increment of 0.2 °C in each cycle. The melting temperature was determined from the first derivative peaks of the melting curve using Bio-Rad CFX96 manager software. Further, the dissociation constant of the complex between FKBP51 and C90 was determined by fitting the resulting data using single site ligand binding model^35^ with the bottom (T1) and top (T2) temperatures fitting to 43.8 and 48.5 °C, respectively. The concentration of FKBP51 (2 µM) was used as the value of the constant P. The fitting was performed using GraphPad Prism, version 7.02. Additionally, we incubated the FKBP51 with 200 µM of control peptide (DDDDDDDDDD) in a similar way.

### X-ray crystallography

#### Protein Crystallization and x-ray Data Collection

The FKBP51 apo- crystals were obtained by vapor-diffusion sitting drop method. 300 nL of 10 mg/mL protein solution was mixed with 18%w/v PEG MME 5 K, 0.2 M NH_4_SO_4_, 0.1 M MES pH 6, using TTP Labtech’s mosquito LCP. The best crystals were soaked with 10 mM of C90 peptide and stored in liquid N_2_. Diffraction data were collected at 100 K at beamline BL14.1 of BESSY^49^, Berlin, equipped with Dectris Pilatus 6 M detector. The diffraction images were integrated and scaled using XDS. Since the diffraction pattern was clearly anisotropic we decided to truncate the reflections in an elliptical fashion by first using the diffraction anisotropy server at the Molecular Biology Institute at UCLA^50^ (http://services.mbi.ucla.edu/anisoscale) and later the more recent autoPROC software^51^ including Staraniso^52^ in development by Global Phasing Ltd. Crystal parameters and data collection statistics for the complex is summarized in Table 1.

#### Structure Determination, Refinement, and Analysis

The structure of FKBP51-Hsp90 C-terminal peptide complex was solved by molecular replacement in Phaser^53^ available in the CCP4 software suite^54^ by using the FKBP51 structure (PDB ID: 1KT0) as search model. Initial rigid-body refinement and subsequent restrained refinement were performed by using BUSTER^55^. The Coot program^37^ was used for manual model building, and addition of water molecules. Final refinements were carried out using BUSTER. We use MolProbity^56^ to monitor refinement progress and finding local errors in our intermediate models. The final refinement statistics are summarized in Table 1. Figures showing structural representations were prepared using PyMOL^57^ and VASCO^58^ for generating electrostatic and hydrophobic surfaces. The simulated annealing omit map was generated in Phenix^59^ by removing the peptide and running the simulated annealing refinement job and the resulting difference density map contoured at 3 σ is shown in Fig. 5B.

### Validation of the crystal structure

The Ramachandran plot was obtained using Rampage Software on the webserver (http://mordred.bioc.cam.ac.uk/~rapper/rampage.php)^40^. The crystal structure of the complex (5NJX) had its quality validated using QMEAN server^41^ and ProSA web server^42^.

### Conservation profile of TPRs

ConSurf server (http://consurf.tau.ac.il/2016/)^60^, was used to carry out the evolutionary conservation. HOP (1ELR)^2^, HOP (1ELW)^2^, FKBP52 (1QZ2)^29^, CHIP (2C2L)^61^, HOP (3ESK)^62^, CHIP (3Q47)^63^, CHIP (3Q49)^63^, AIP (4AIF)^31^, AIP (4APO)^31^, CHIP (4KBQ)^64^, FKBP38 (5MGX)^30^, FKBP51 (5NJX) and Tah1 (4CGQ)^65^ structures were downloaded from the protein data bank (http://www.rcsb.org) and their TPR domain were extracted. The resulting TPR domains were aligned using Theseus^66^, which is a program that uses maximum likelihood super-positioning to align multiple macromolecular structures. The multiple sequence alignment output from Theseus was then used in ConSurf web server to carry out evolutionary conservation of amino acid residues in the TPR domain.

### Molecular dynamics simulations

Molecular dynamics (MD) simulation of the FKBP51 in complex with Hsp90 C-terminal peptide was carried out in order to explore the binding mode of the peptide with the TPR domain of FKBP51. MD simulation was performed using GROMACS 5.1.4 code using Gromos54a7 force field^67–69^. The complex structure obtained from x-ray crystallography containing TPR residues from Met259 to Asp405 with the peptide residues from Asp724 to Asp732 was solvated in a cubic box of 971 nm^3^ using simple point charge (SPC/E) water model keeping a distance of 1.0 nm between each side. The final simulation system was solvated and ionized with Na+ and Cl^−^ ions at a concentration of 100 mM and the final system consisting of 18255 water molecules and 37 Na+ and 35 Cl^−^ ions was minimized using the steepest descent algorithm until the maximum force became less than 500 kJ/mol/nm. In order to avoid the distortion of the system during production simulation, position restrained equilibration run was performed after minimization for 1 ns using constant number, volume and temperature (NVT) and isothermal-isobaric (NPT) ensemble. The length of equilibration step was determined on the basis of convergence of quantitative metrics (temperature, density, etc.) which indicates the state of equilibrium. V-rescale temperature coupling^70^ and Parrinello–Rahman pressure coupling^71^ were applied to maintain the system in at 300 K temperature and 1 bar pressure along with coupling constant of 0.1 picosecond (ps) for temperature and 2 ps for pressure. During the NVT and NPT ensemble simulations, position restraints were applied to the complex. Long-range electrostatic interactions and van der Walls interactions were calculated using the Particle mesh Ewald (PME) method^72^, and the cut-off for short-range van der Waals was set to 1 nm. All bonds were constrained using LINCS algorithm^73^ and the time step of the simulation was set to 0.002 ps. Finally, a 100 ns productions simulation was performed.

### Analysis of molecular dynamics simulation trajectory

The MD trajectory obtained from the simulation was thoroughly analyzed using the functionalities present in GROMACS 5.1.4. Quantitative analysis of the simulation trajectory was performed by determining root means square deviation (RMSD), root mean square fluctuation (RMSF) and radius of gyration (Rg) using rmsd, rmsf, and gyrate functionalities. Structural analysis was performed by analyzing the hydrogen bonds formed during the 100 ns simulation between TPR domain of FKBP51 and C90 peptide using the h-bond utility in GROMACS 5.1.4.

### Accession numbers

The coordinates and structure factors of the solved crystal structures for the native and FKBP51-Hsp90 C-terminal peptide complex have been deposited in the Protein Data Bank with accession numbers 5OMP and 5NJX, respectively.
